# Integrative analysis of the molecular signature of target genes involved in the antitumor effects of cantharidin on hepatocellular carcinoma

**DOI:** 10.1186/s12885-023-11594-8

**Published:** 2023-11-28

**Authors:** Jia Yan, Yu min Gao, Xiu ling Deng, Hai sheng Wang, Gui tao Shi

**Affiliations:** 1 School of Basic medical, Inner Mongolia Medical University, Hohhot, Inner Mongolia China; 2 School of Public health, Inner Mongolia Medical University, Hohhot, Inner Mongolia China; 3 Affiliated Hospital of Inner Mongolia Medical University, Hohhot, Inner Mongolia China

**Keywords:** Cantharidin, Hepatocellular carcinoma, Autophagy, Immune response, Fatty acid metabolism

## Abstract

**Background:**

Cantharidin (CTD) is the active ingredient of Chinese medicine, which has been traditionally used in multiple cancers treatment, especially in hepatocellular carcinoma (HCC). However, a comprehensive analysis of the CTD-related molecular mechanism is still necessary to understand its functions in HCC treatment. This study aimed to reveal the novel molecular targets and regulatory networks of CTD in HCC.

**Methods:**

A model of H22 tumour-bearing mice was constructed, and the function of CTD in tumour growth was evaluated. An integrated approach of CTD associated transcriptional profiling and biological systems analysis was used to identify key regulators involved in antitumour pathways. The identified differential expression patterns were supported by the results of Gene Ontology (GO) term and Kyoto Encyclopedia of Genes and Genomes (KEGG) pathway enrichment analyse, and by protein-protein interaction (PPI) network construction. The relationships between gene expression and tumour immunity were evaluated using Tumour Immune Estimation Resource (TIMER). Prognostic value was analyzed with Kaplan-Meier plotter.

**Results:**

In the present study, the therapeutic effect of CTD on HCC was evaluated *in vivo.* We obtained the CTD-related transcriptional profiles, systematically and intuitively illustrated its possible pharmacological mechanisms in HCC through multiple targets and signalling pathways. These results revealed that the CTD-related differentially expressed genes were involved in autophagy, transcription factors (TFs) related transcriptional regulation, fatty acid metabolism and immune response in HCC. We found that *MAPT*, *TOP2A, CENPF* and *MEFV* were hub genes of CTD targets involved in autophagy regulation. Totally, 14 TFs have been confirmed to be critical for transcriptional regulation, and 33 TF targets were identified as the hub genes in transcriptional mis-regulation pathway in cancer. These TFs were associated with the immune response and immune cell infiltration. In addition, the downregulated genes were significantly enriched in metabolic regulation pathways, especially fatty acid metabolism after CTD treatment. Furthermore, the network of CTD associated miRNAs with these fatty acid metabolism-related targets was constructed in HCC.

**Conclusions:**

Taken together, our results comprehensively elucidated that CTD could act on multiple targets in HCC therapy, affecting autophagy, transcriptional regulation, the immune response and fatty acid metabolism. Our results provide a foundation for the study of the molecular mechanistic of CTD and its clinical application in the treatment of HCC.

**Supplementary Information:**

The online version contains supplementary material available at 10.1186/s12885-023-11594-8.

## Introduction

Liver cancer is one of the most common malignant tumours, and its prevalence and death rates have risen worldwide in recent years [1]. Statistics showed that 90% of liver cancers are hepatocellular carcinomas (HCCs) [1]. Traditional therapies for HCC include surgery, chemotherapy, radiotherapy, targeted therapy, and immunotherapy. However, the cure rate is still very low, since a majority of patients present with advanced disease. In recent years, traditional Chinese medicine (TCM) has shown obvious advantages in liver cancer treatment, acting as a multi-target adjuvant therapy [1, 2]. Combining TCM with conventional therapeutic drugs is anticipated to increase sensitivity to effectively inhibit the development of liver cancer [1]. It has been reported that this approach could increase sensitivity to ameliorate adverse effects of chemotherapy and targeted therapy in patients with HCC [3].

Cantharidin (CTD) is a main active component in TCM, which is a bioactive sesquiterpenoid isolated from insects of the genus *Mylabris.* It has been reported that CTD shows obvious cytotoxic activity towards cancer cell to suppress carcinoma cell growth and proliferation in a time- and dose-dependent manner, especially in liver cancer [4]. However, its potential preclinical application is limited by its toxicity. It has been reported that CTD is an inhibitor of protein phosphatase 1 (PP1) and protein phosphatase type 2 A (PP2A), that could induce cell apoptosis and effect protein synthesis [5]. CTD can accelerate apoptosis in different cancer cell lines via the intrinsic and extrinsic apoptotic pathways or the endoplasmic reticulum pathway [6]. It has also been reported that CTD could increase the expression of death-related genes, including *DR5*, *PUMA*, *BTG2*,* NOXA*, *GADD45* and* TRB3* to induce the activation of extrinsic apoptosis pathway [7–9]. It promotes *ATF6β*,* IRE1α*, *IRE1β*, *GRP78*, *caspase-4*, *calpain-2* and *XBP-1* expression to induce apoptosis via the ER stress associated pathway [10–13]. In addition, CTD also effectively induce autophagy by modulating the level of several autophagy associated proteins, including Beclin-1, LC3-I/LC3-II, and PI3K/AKT/mTOR signalling pathway proteins [14–16]. Therefore, CTD is responsible for tumour destruction through apoptosis, necrosis and autophagy.

Despite the remarkable therapeutic efficacy of CTD in liver cancer, the understanding of its molecular mechanism is still limited. It has been reported that CTD inhibits HCC development through p38 MAPK, JAK2/STAT3, PI3K/Akt and LC3 related autophagy pathways [16–18]. It induces DNA damage via KDM4A-dependent H3K36 methylation [19]. However, the severe toxicity of CTD prevents its clinical application. Therefore, its regulatory mechanisms in HCC are still unclear. An in-depth study of CTD-related molecular regulation is not only helpful for revealing its anticancer activity but also helpful for analysing its cytotoxicity at the cellular level.

In this study, we integrated transcriptional profiling and network analysis to identify potential biomarkers and targets of CTD in HCC cells. We investigated CTD associated autophagy, transcriptional regulation, immune responses and fatty acid metabolism in HCC. The novel potential targets and their regulatory networks were confirmed in HCC. Moreover, the relationships of these hub genes with tumour immune infiltration were evaluated. CTD-related miRNAs that target fatty acid metabolism-related genes were assessed, and a fatty acid-related miRNA-target networks were constructed.

## Results

### Cantharidin inhibits HCC tumour growth in vivo

**Fig. 1:**
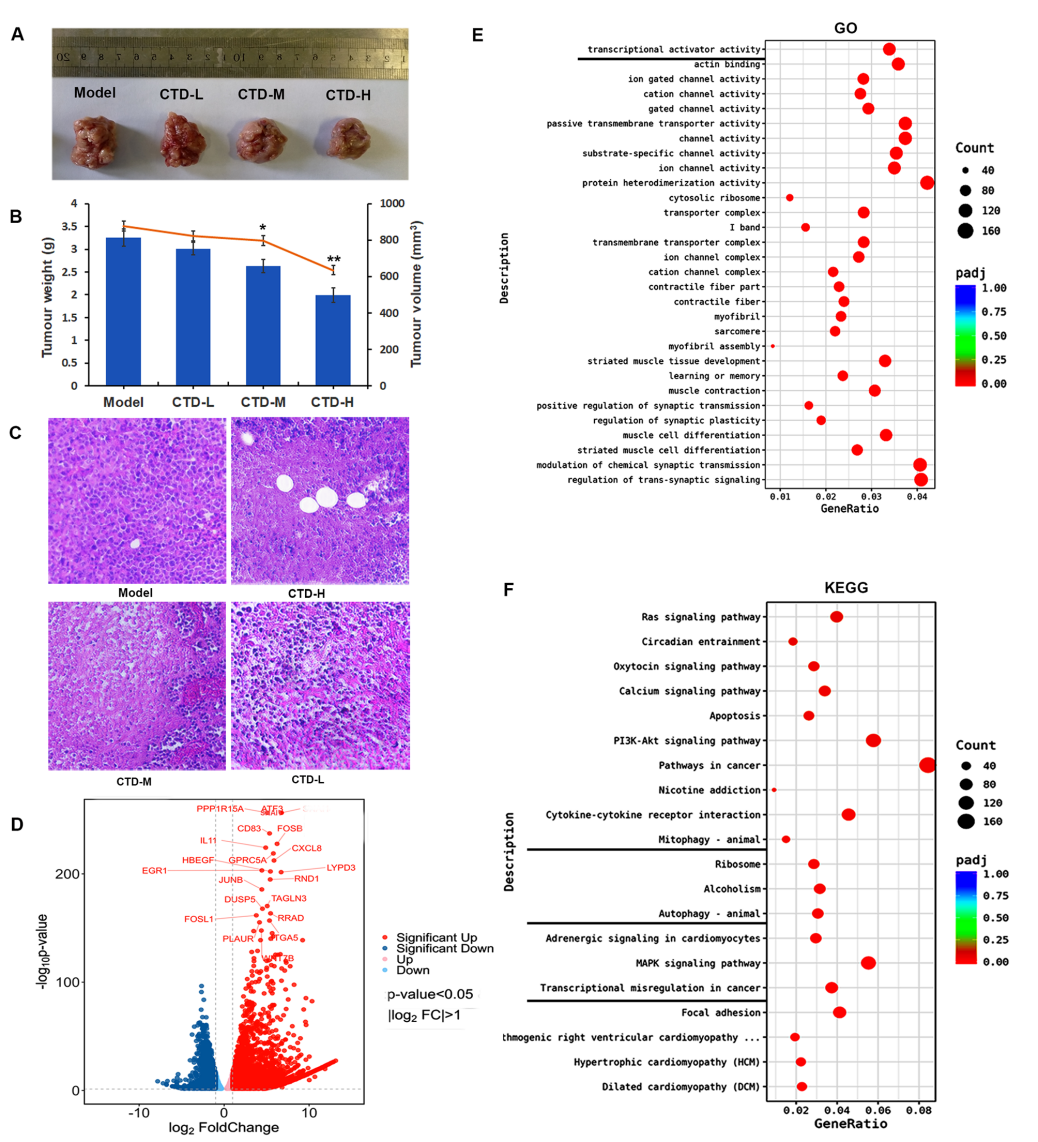
The cantharidin inhibits the tumor growth in mice. (**A, B**) Cantharidin represses the tumor cell growth *in vivo.* The tumor volume (**A**) and weight (**B**) were shown. Data were presented as the mean ± SD (n = 3), * *p* < 0.05 vs. the control group. (**C**) The H&E staining results of tumor tissues, including model, CTD high, middle, and low groups. The tumor tissues from model were acted as control, and the tumor tissues of CTD treatment were treated groups. (**D**) The volcano plot showed the differentially expressed genes between cantharidin treated groups and control group. The upregulated genes were shown by red color; the downregulated genes were shown by blue color. (**E, F**) The function enrichment analysis of CTD-associated differentially expressed genes was showed by (**D**) GO and (**E**) KEGG enrichment

To confirm the antitumour function of CTD in HCC, we investigated the effect of CTD on tumour growth *in vivo.* The HCC mouse model was constructed based on H22 cells and the antitumour effect of CTD was evaluated. The chemical formula of CTD is shown the supplementary Figure S1. After CTD treatment, the volume and weight of tumour tissues were significantly decreased, the tumor weight was 1.99 g and 2.63 g, and the volume was 635 mm^3^and 798 mm^3^ in mouse treated with a high- and middle-doses of CTD, respectively (Fig. 1A, B). Tumor growth inhibition rates were 38.96% and 19.33% in high- and middle-doses groups, respectively. While, the effect of inhibiting tumor growth was not significant in the low dose group. Furthermore, the HE staining results showed that the number of cells was significantly reduced, and that these cells were loosely arranged in tumuor tissues. The necrotic and apoptotic cells were significantly increased in the CTD group, with increase in the number of abnormal vacuoles in the middle and high CTD concentration groups (Fig. 1C). These results indicated that CTD could inhibit tumour cell growth and promote cellular necrosis *in vivo.*

### CTD induces apoptosis and autophagy to affect the growth of HCC cells

To further explore the novel therapeutic targets and potential mechanism of CTD in HCC, RNA-seq analysis was performed. Based on a threshold value of *p *value < 0.05 and |log2FoldChange| > 1, total of 7008 genes were significantly upregulated and 4973 genes were significantly downregulated in the cantharidin treated group (Fig. 1D). Subsequently, GO and KEGG enrichment analyses were performed. The Go analysis results showed that the upregulated DEGs were significantly enriched in the terms transcriptional activator activity, protein heterodimerization activity, modulation of chemical synaptic transmission and trans-synaptic terms (Fig. 1E). Next, KEGG enrichment analysis was performed, and the results showed that pathways in caner, PI3K-AKT, MAPK and RAS signaling pathways were enriched in the DEGs after CTD treatment. Moreover, these DEGs were also involved in apoptosis, autophagy and mitophagy (Fig. 1F). Therefore, CTD might affect cellular apoptosis by regulating the autophagy and mitophagy pathways in HCC.

**Fig. 2:**
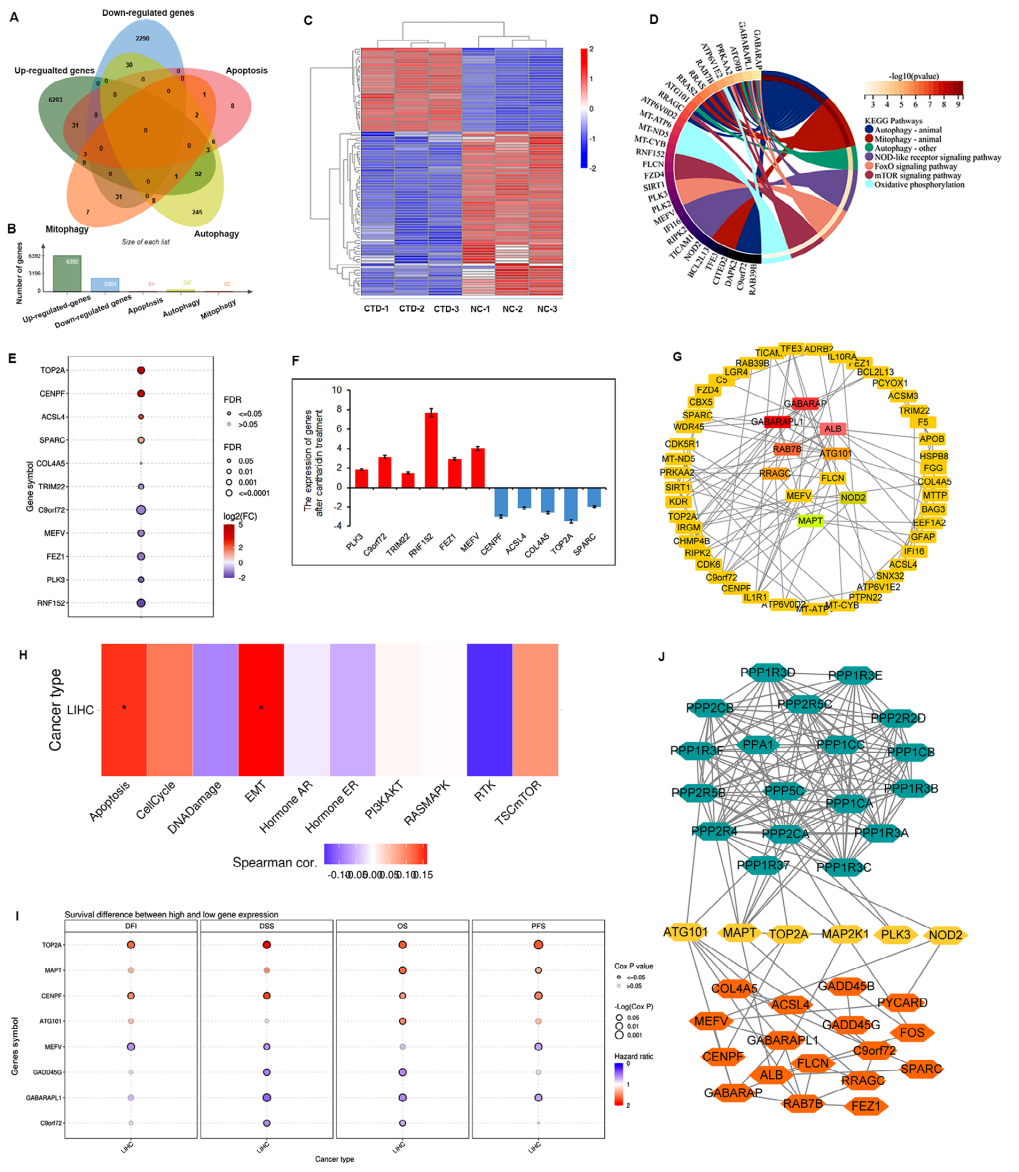
The cantharidin related autophagy targets in HCC. (**A**) The venn diagram showed the apoptosis-, autophagy- and mitophagy-related DEGs after CTD treatment. (**B**) The number of genes in different groups. (**C**) Heat map showed the expression of theses genes. The upregulated genes were indicated by red color; the downregulated genes were indicated by blue color. (**D**) The network between the autophagy- and mitophagy-related DEGs and their involved functional pathways were drawn based on the KEGG analysis results. (**E**) The expression of autophagy-related DEGs of CTD in liver cancer. (**F**) The expression of autophagy-related DEGs were detected by RT-PCR in CTD treated HepG2 cell. The upregulated genes were indicated by red color; the downregulated genes were indicated by blue color. *p* < 0.05 was considered the significant difference. (**G**) The protein interaction network of autophagy-related DEGs after CTD treatment. The top ten hub genes were shown inside the circle. Red to green color indicated a correlation trend. Red color indicated the highest correlation. (**H**) The correlations between CTD regulated autophagy-related DEGs and different functional pathways in liver cancer. (**I**) Kaplan–Meier analysis of the relationship between hub genes and OS in patients with liver cancer. (**J**) A protein interaction network of phosphatases family proteins with CTD regulated autophagy- related hub genes

Furthermore, apoptosis-, autophagy- and mitophagy-related gene sets were obtained from MSigDB. Venn diagram analysis was used to confirm the DEGs, in the apoptosis, autophagy and mitophagy pathways. In total, after CTD treatment, 31 apoptosis-related, 52 autophagy-related and 9 mitophagy-related genes were upregulated, while 30 autophagy-related and 31 mitophagy-related genes were downregulated (Fig. 2A, B). The heatmap of these DEGs was showed in Fig. 2C. Furthermore, KEGG enrichment analysis of these DEGs was performed and showed that these genes were also involved in NOD-like receptor, FoxO, and mTOR signalling pathways, and in oxidative phosphorylation processes (Fig. 2D).

We further conducted an in-depth analysis of the function of these DEGs in HCC. As shown in Fig. 2E, *TOP2A*, *CENPF*, *ACSL4*, *SPARC*, and *COL4A5* were significantly upregulated in patients with liver cancer, while they were downregulated by CTD treatment in HCC cells. *PLK3*, *C9orf72*, *TRIM22*, *RNF152*, *FEZ1* and *MEFV* were dramatically downregulated in liver cancer patients, but upregulated by CTD treatment in HCC cells (Fig. 2F). Additionally, a protein interaction network was constructed based on these autophagy-related gene set regulated by CTD. The results indicated that *GABARAP*, *GABARAPL1*, *RAB7B*, *ALB*, *ATG101*, *RRAGC*, *FLCN*, *MEFV*, *MAPT* were the top 10 hub genes in CTD regulated autophagy network (Fig. 2G). GSVA suggested that these hub genes also have significant relationships with the apoptosis and EMT pathways in HCC (Fig. 2H). Moreover, we found that hub genes, *TOP2A*, *MAPT*, *CENPF*, *ATG101*, *MEFV*, *GADD45G*, *GABARAPL1* and *C9orf72* were significantly associated with a worse prognosis in patients with HCC (Fig. 2I).

Because CTD acts as a protein phosphatases inhibitor, we obtained phosphatases family proteins PP1, PP2A and PP5 and constructed a protein interaction network with the CTD-related hub genes. We found that ATG101, MAPT, TOP2A, MAP2K1, PLK3 and NOD2 directly interact with phosphatases to combine with other autophagy-related genes to induce apoptosis in HCC cells (Fig. 2J). Therefore, *MAPT*, *TOP2A, CENPF* and *MEFV* were identified as hub genes of CTD targets that regulate autophagy and also sever as oncogenes or tumour suppressor genes to affect the prognosis of patients with liver cancer.

### CTD is involved in transcriptional regulation in HCC

**Fig. 3:**
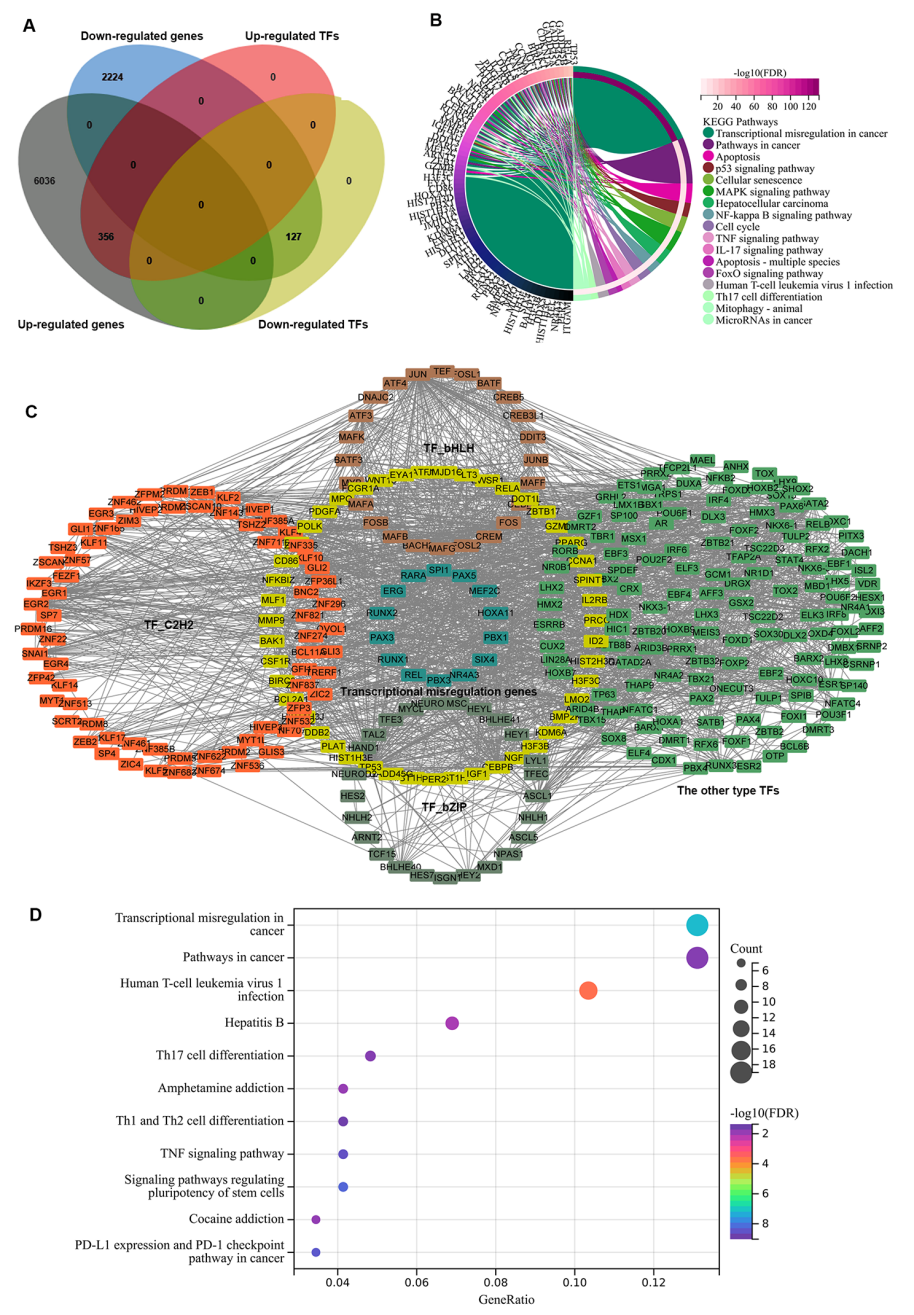
The transcriptional mis-regulation involved targets of cantharidin in HCC. (**A**) The venn diagram showed the different expression of TFs after cantharidin treatment. (**B**) The pathways that were associated with the cantharidin regulated TFs in HCC based on the KEGG results. (**C**) The protein interaction network of cantharidin regulated TFs that were involved in transcriptional misregulation after cantharidin treatment. Yellow color indicates the DEGs that were involved in transcriptional misregulation. Dark green indicates the hub TFs of this network. The orange color indicated C2H2 family, the brown color indicated bHLH family, turquoise color indicated bZIP family, green color indicated the other type TFs. (**D**) The KEGG enrichment analysis result of CTD-regulated hub TFs and genes were involved in transcriptional mis-regulation pathway in cancer

Because the KEGG analysis results indicated that the transcriptional mis-regulated in cancer pathway was enriched in the CTD regulated DEGs, we focused on CTD-associated differentially expressed TFs in HCC. In total, 356 upregulated and 127 downregulated TFs were identified in CTD treated HCC cells (Fig. 3A). KEGG analysis showed that the upregulated TFs were involved in the transcriptional misregulation in cancer. As shown in Fig. 3B, these TFs were associated with pathways in cancer, including the apoptosis, cellular senescence, mitophagy, cell cycle and miRNAs in cancer pathways. Moreover, they were also involved in the regulation of multiple tumour-related signalling pathways, such as p53, MAPK, NF-kappa B, TNF, and IL-17 signalling pathways in HCC.

Furthermore, the protein interaction network of these TFs was constructed based on the STRING database. We classified these CTD-regulated transcription factors and found that the top three TF families were C2H2, bHLH, and bZIP family. As shown in Fig. 3C, the transcription factors subset, which were directly involved in the transcriptional misregulation pathway, were marked in yellow and dark green. Among them, the CTD regulated hub TFs were  *SPI1*, *PAX5*, *MEF2C*, *HOXA11*, *PBX1*, *SIX4*, *NR4A3*, *PBX3*, *REL*, *RUNX1*, *PAX3*, *RUNX2*, *ERG*, and *RARA*. (Fig. 3C). In addition, GO enrichment analysis indicated that these hub TFs were enriched in the Th17, Th1 and Th1 cell differentiation pathways and in the PD-L1 expression and PD-1 checkpoint pathway. These results suggested that CTD-targeted TFs likely participate in the regulation of anti-tumor immune responses in HCC (Fig. 3D).

### The effects of cantharidin on immune responses in HCC

**Fig. 4:**
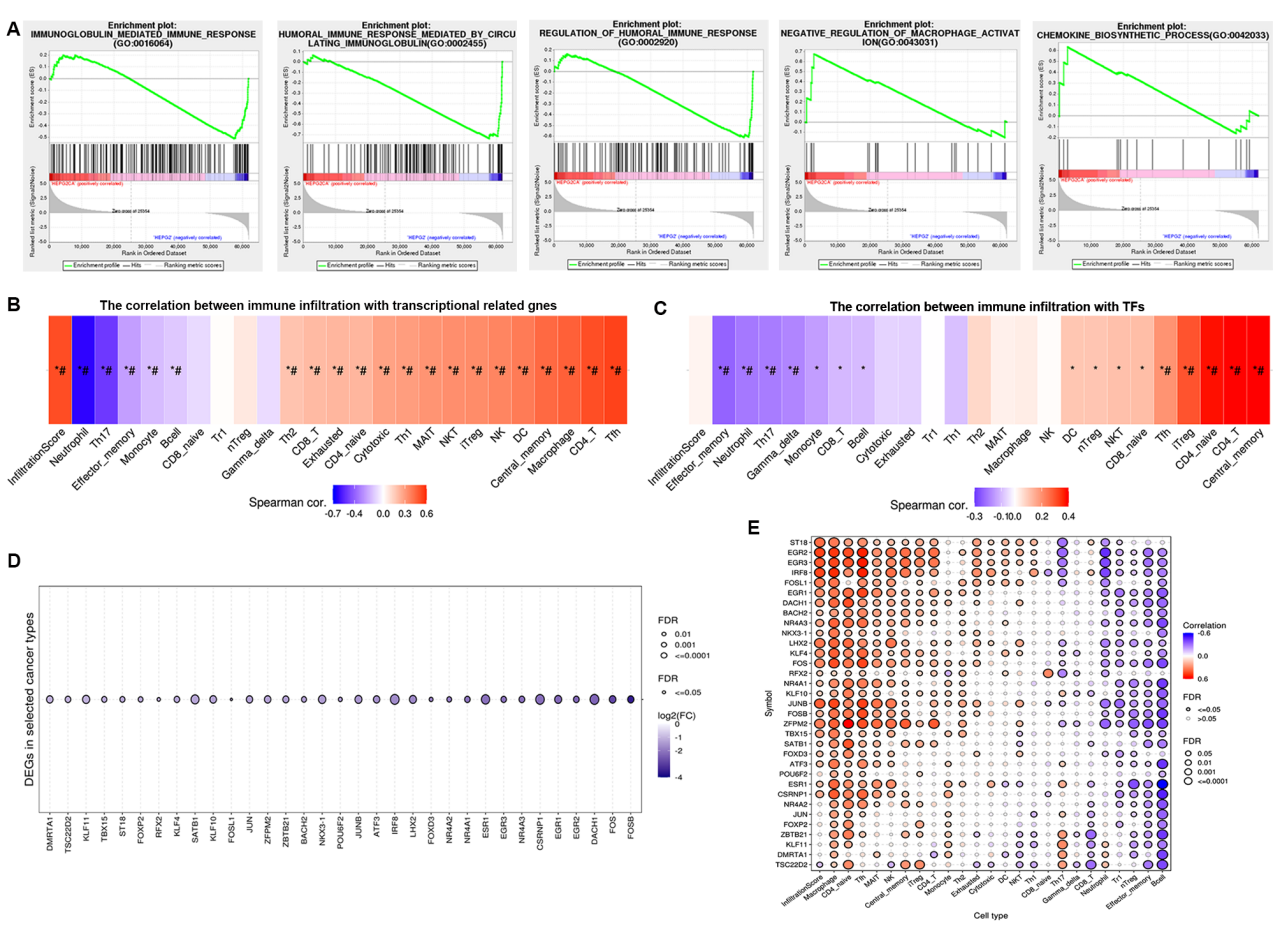
The cantharidin associated TFs were involved in immune response. (**A**) GO functional annotation showed that cantharidin regulated DEGs were positively associated with immune response, macrophage activation and chemokine biosynthetic processes based on GSEA analysis. (**B, C**) The heatmap showed the correlation between immune infiltration and CTD-regulated transcriptional genes (**B**) and hub TF genes (**C**). (**D**) The heatmap showed the CTD regulated DEGs and its expression was decreased in LIHC patients based on TCGA data. (**E**) The correlation between immune infiltration and these down-regulated genes in LIHC. The immune infiltration was analyzed using the GSCA tools. Red color indicated positive correlation; bule color indicated negative correlation. * *P* < 0.05; # FDR < 0.05

To further insight into the expression of CTD-related genes involved in signalling pathways in HCC, GSEA was performed. The results showed that CTD-related genes were positively associated with the terms related to immune responses in HCC, including immunoglobulin mediated immune response, humoral immune response mediated by circulating immunoglobulin, regulation of humoral immune response, negative regulation of macrophage activation, and chemokine biosynthetic process (Fig. 4A). In additions, the association results indicated that the CTD-regulated genes, which were involved in the transcriptional misregulation, were dramatically associated with the infiltration of immune cells in liver cancer. The infiltration score showed that the expression of these genes was positively associated with the infiltration of immune cells, including Tfh, CD4 + T cells, macrophages, and DC cells, while negatively associated with the infiltration of neutrophils and Th17 cells (Fig. 4B). Furthermore, the relationships between the CTD-related differentially expressed TFs and the infiltration of immune cells were also evaluated. The results suggested that the expression of these TFs was significantly positively associated with the infiltration of immune cells, including central memory cells, CD4 + T cells, CD4 + naïve T cells and iTreg cells (Fig. 4C). Furthermore, we assessed the expression of these immune-related genes in liver cancer based on the TCGA database, and identified 33 genes were significantly downregulated in liver cancer (Fig. 4D). Moreover, the infiltration score also indicated that their expression was significantly associated with infiltration of CD4 + T cells and macrophages, and negatively correlated with infiltration of B cells and effector memory cells (Fig. 4E). Considering these results collectively, we speculated that CTD likely affects antitumour immune response by upregulating the expression of these TFs to activate immune response pathways and induce the infiltration of immune cells in HCC.

### CTD regulates metabolic pathways in HCC

**Fig. 5:**
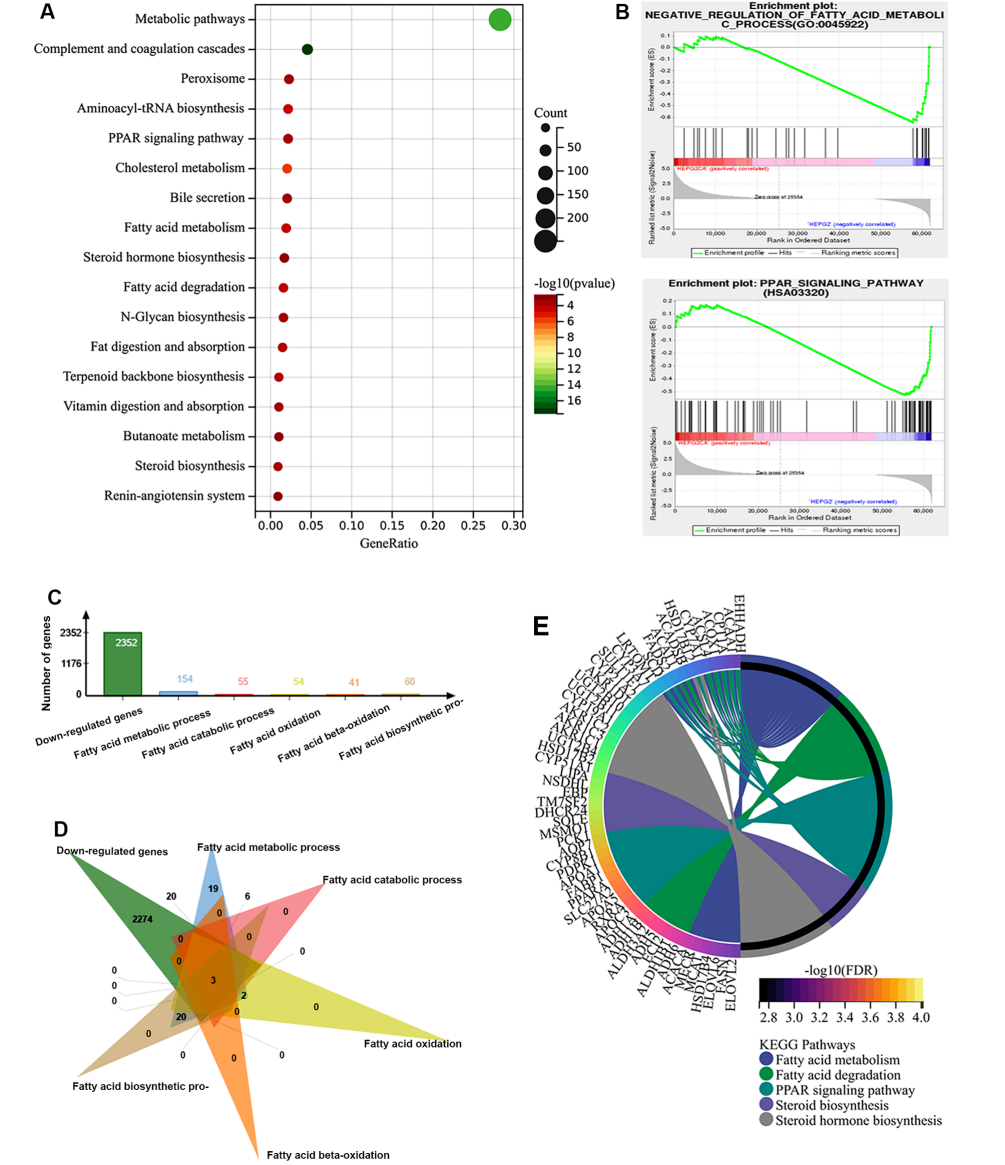
Cantharidin down-regulated genes were involved in fatty acid metabolism. (**A**) The KEGG enrichment analysis indicated that CTD down regulated genes were involved in metabolism pathways, including fatty acid and steroid. (**B**) CTD downregulated genes were positively associated with fatty acid metabolic process and PPAR signaling pathway based on GSEA analysis. (**C**) Statistical analysis showed the fatty acid metabolism-related gene sets from the MSigDB. (**D**) The venn diagram showed the overlap between CTD down regulated gene set and fatty acid metabolism-related gene sets. (**E**) The network was shown the relations between CTD down regulated genes and fatty acid and steroid metabolic processes pathways

To further evaluate the mechanism of CTD in HCC therapy, GO enrichment analysis of genes down-regulated by CTD was examined, and the results showed that they were associated with metabolic pathways (Fig. 5A). Moreover, GSEA showed that they were significantly involved in the negative regulation of fatty acid metabolic processes and the PPAR signalling pathway (Fig. 5B), suggesting that CTD has advantages in ameliorating cancer cell metabolic reprogramming, especially fatty acid metabolic regulation, in HCC. In addition, 154 the fatty acid metabolism-, 55 fatty acids catabolic-, 54 fatty acid oxidation-, 41 fatty acid beta-oxidation-, and 60 fatty acid biosynthetic-related genes were obtained from MigBD (Fig. 5C). The venn diagram showed that totally 34 fatty acid metabolic processes related genes were downregulated by CTD (Fig. 5D). Furthermore, the network of fatty acid- related genes down regulated by CTD with metabolic pathways was confirmed based on KEGG analysis (Fig. 5E).

**Fig. 6:**
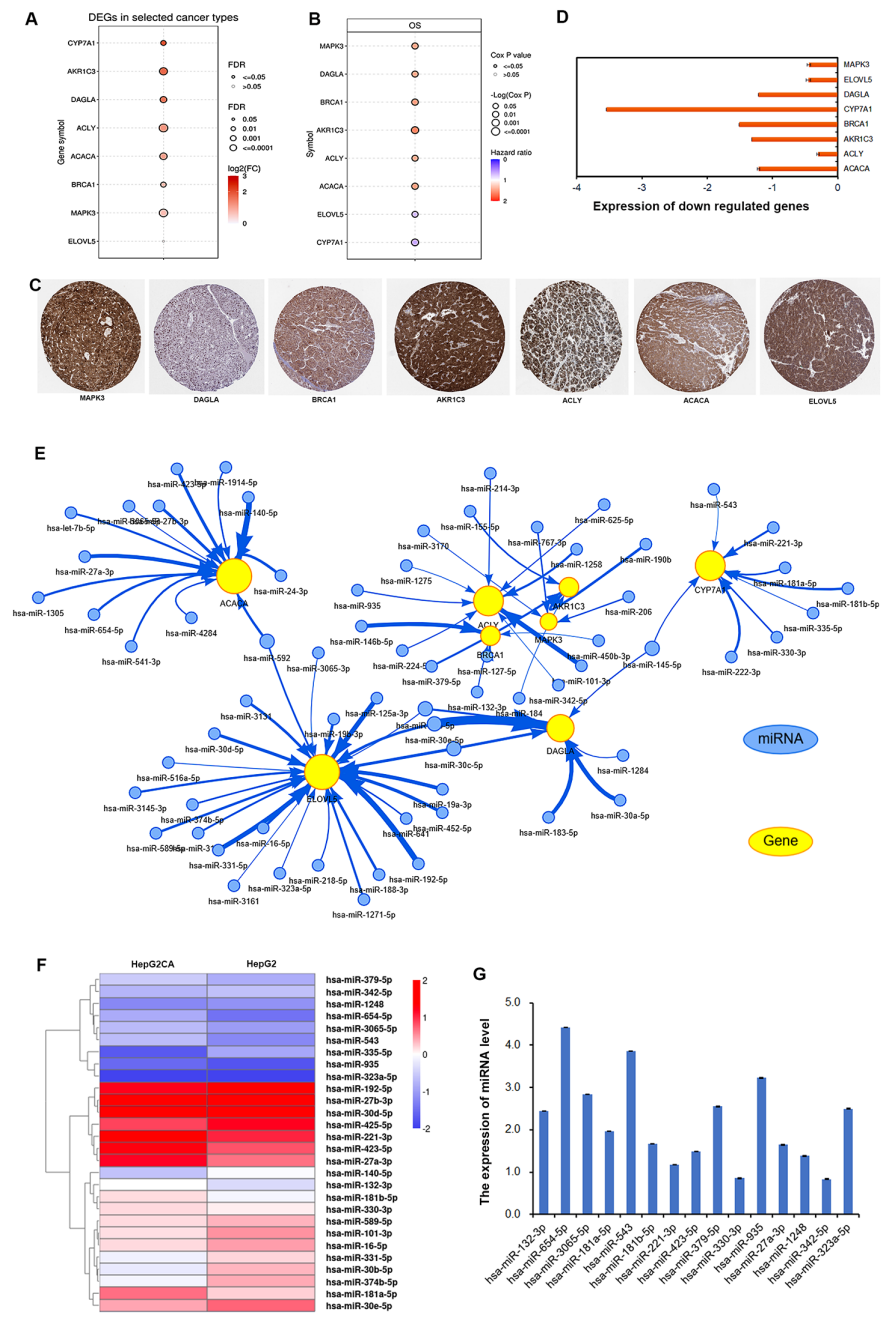
Cantharidin regulated miRNA to suppress the expression of genes related to fatty acid metabolism. (**A**) The CTD downregulated fatty acid metabolism-related gene were elevated in LIHC. The heatmap showed the expression of these genes in LIHC. (**B**) The genes indicated poor prognosis and involved in fatty acid metabolic process were confirmed. The red color indicated positive correlation; bule color indicated negative correlation. (**C**) The expressions of key genes related to fatty acid metabolism were assessed by RT-PCR in CTD treated HepG2 cell. (**D**) The protein level of genes related to fatty acid metabolism in patients with HCC. (**E**) The network of miRNAs targeting the key fatty acid metabolism related genes after CTD treatment. (**F**) The heatmap showed the predicted miRNA genes expression in CTD treated HepG2 cells. (**G**) The expression of CTD upregulated miRNAs related to fatty acid metabolism in HCC cells

To confirm the hub genes of CTD-targets about fatty acid metabolism regulation in HCC, we assessed the expression of these genes in the LIHC dataset in TCGA. We found that the expression levels of eight genes, namely, *CYP7A1*, *AKR1C3*, *DAGLA*, *ACLY, ACACA*, *BRCA1*, *MAPK3*, and *ELOVL5* were increased in LIHC and that their upregulation were indicated a poor prognosis in patients with HCC (Fig. 6A, B). Moreover, their protein levels also dramatically elevated in LIHC patients (Fig. 6C). However, their expression levels were significantly decreased after CTD treatment (Fig. 6D). In addition, we explored the reason for affecting the expression of these genes. We focused on the regulation of miRNA during antitumor therapy of CTD in liver cancer. A network between fatty acid metabolism-related hub genes and miRNAs was constructed (Fig. 6E). The expression of miRNAs in this network was elevated after CTD treatment in HCC. The corresponding expression heatmap was shown in Fig. 6F. Among these miRNAs, the miRNAs with upregulated expression were selected and verified for expression after CTD treatment. A total of 8 upregulated miRNAs were identified (Fig. 6G). Taken together, CTD-upregulated miRNAs and their targets were identified, including miR654-5p/miR423-5p/miR27a-3p/*ACACA*, miR132-3p/miR3065-5p/miR323-5p/*ELOVL5*, miR132-3p/*DAGLA*, miR181a/b-5p/miR543/miR221-3p miR330-3p/*CYP7A1*, miR935/*ACLY*, miR342-5p/*MAPK3*, miR379-5p/*BRCA1*, which were involved in fatty acid metabolism pathway. Therefore, CTD may target these miRNAs to suppress the expression of fatty acid metabolism-related hub genes to inhibit tumour cell growth in HCC.

## Discussion

HCC has become one of the leading causes of cancer-related death worldwide due to its heterogeneity and dismal prognosis. In recent years, traditional Chinese medicines, particularly formulas, have demonstrated obvious advantages in the treatment of liver cancer. These benefits involve multiple targets and biological pathways. In this study, the transcriptional regulatory effects of cantharidin in HCC cells were analysed comprehensively. This study not only provides a foundation for molecular mechanistic research of the anti-tumour effective of CTD in HCC but also beneficial for understanding the cytotoxicity of CTD in liver cells.

Accumulating evidence suggests that inhibiting autophagy can induce apoptosis during anticancer therapy. It has been confirmed taht sodium cantharidate could induce apoptosis and autophagy by inhibiting the PI3K-Akt-mTOR pathway [15]. NCTD-induced apoptosis is enhanced by ATG5 and LC3, which were associated with autophagy pathway in HCC [20, 21]. Our results also showed that CTD was involved in inhibiting autophagy to induce apoptosis in HCC cells. However, we found several novel targets of CTD related to autophagy that had not been previously reported, such as *GABARAP*, *GABARAPL1*, *ALB*, *RAB7B*, *ATG101*, *GLCN*, *MEFV*, *NOD*, and *MAPT*. Moreover, we confirmed that the CTD-downregulated genes *TOP2A*, *CENPF*, *ACSL4*, *SPARC*, and *COL4A5* were significantly increased in liver cancer patients, while upregulated genes *PLK3*, *C9orf72*, *TRIM22*, *RNF152*, *FEZ1* and *MEFV* were dramatically decreased in liver cancer patients. Among them, *TOP2A*, *CENPF*, *MEFV *and *C9orf72* directly affect the prognosis of patients with liver cancer. Therefore, we speculated that these genes are likely the direct antitumor targets of CTD resulting in autophagy inhibition induced apoptosis.

It has been reported that the anticancer mechanisms of CTD are associated with its activity as an inhibitor of protein phosphatases (PP), including PP1, PP2A and PP5, to induce the formation of DNA strand breaks and cell apoptosis [22–24]. Moreover, phosphatases play an important role in the regulation of autophagy or mitophagy, and it has identified that many of the core autophagy proteins are as the direct targets of phosphatases [25, 26]. In this study, we further integrate analyzed the network between protein phosphatases and CTD-related autophagy gene set. The hub genes of CTD-related autophagy *ATG101*, *MAPT*, *TOP2A*, *MAP2K1*, *PLK3* and *NOD2* were confirmed that could directly interact with phosphatases PP1, PP2A and PP5. We speculated that CTD targets these genes through phosphatases to induce apoptosis or autophagy, which will contribute to the treatment of HCC and exert the best antitumour effect in HCC.

It is well known that the inhibitory effects of traditional Chinese medicines and natural medicines on tumours are reflected at multiple levels and in multiple pathways. While, transcription factors (TFs) are key regulators of cellular processes [27]. Therefore, the antitumor function of CTD involving multiple signaling pathways may be attributed to its targeting of numerous TFs. We constructed a CTD-dependent TF interaction network and found that these hub genes in CTD-related transcriptional regulation were *SPI*, *PAX5*, *MEF2C*, *HOXA11*, *PBX1*, *SIX4*, *NR4A3*, *PBX3*, *REL*, *RUNX1*, *PAX3*, *RUNX2*, *ERG*, and *RARA*. In addition, these TFs were involved in immune response- and immunotherapy-related pathways. Considering these results collectively, we speculated that CTD inhibits tumour progression by integrating multiple transcription factor networks related signaling pathways.

**Fig. 7:**
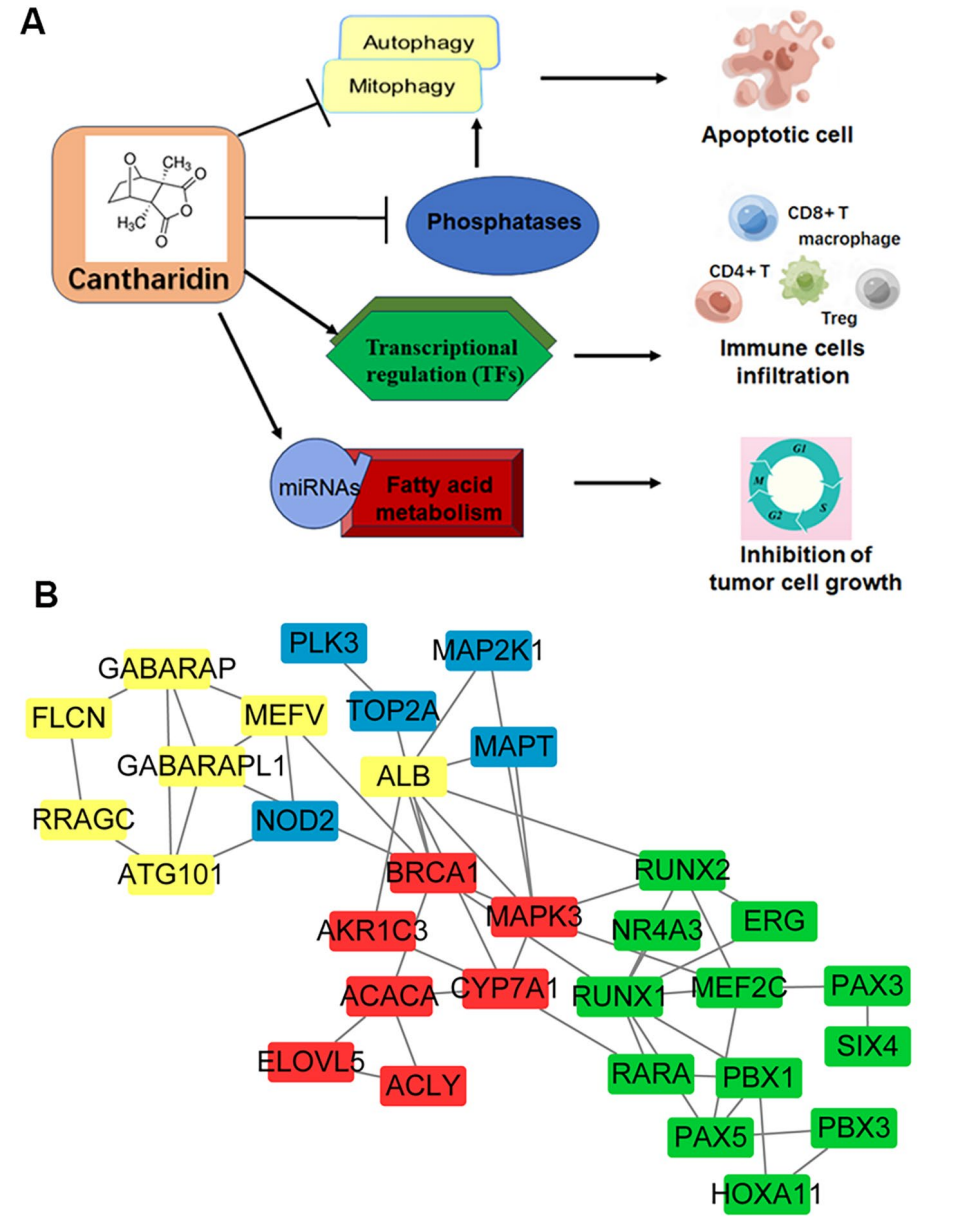
Cantharidin inhibits tumor growth by integrating multiple pathway networks. (**A**) Cantharidin inhibits tumor growth by regulating autophagy or mitophagy, TF-related transcriptional regulation, and fatty acid metabolism pathways. (**B**) The molecular regulatory network of CTD function related hub genes in above pathways. Yellow and blue showed autophagy- or mitophagy-related genes; blue color showed genes could directly interact with phosphatases; red color represented fatty acid metabolism related genes; green color indicated TFs

Studies have shown that traditional Chinese medicine has antitumor activity by inhibiting the overexpression of immune checkpoint molecules and enhancing the efficacy of tumor immunotherapy [28]. Therefore, the regulatory function of CTD on tumour immunity cannot be ignored. However, the molecular mechanism of CTD in tumour infiltrating lymphocytes remains unelucidated. In this study, the GSEA results indicated that the DEGs after CTD treatment were involved in immune responses and in macrophage and chemokine biosynthetic processes. Moreover, CTD-related TFs were associated with immune cell infiltration, especially CD4 + T cells, Treg cells and neutrophil infiltration. In particular, the significantly upregulated oncogenes after CTD treatment were correlated with the infiltration of macrophages, CD4 T cells and B cells. This is consistent with previously reported results that cantharidin combination therapy will promote increases in CD8 + T cells and CD4 + Teff cells among tumour infiltrating lymphocytes to inhibit lung cancer growth [29]. CTD likely promotes the activation of transcription genes that plays a role in antitumour immune activation in liver cancer. The liver is a major organ for fatty acid metabolism and synthesis. It has been confirmed that fatty acid metabolism is critically involved in the progression of multiple cancers and the hepatotoxicity of drugs, especially traditional Chinese medicines [30–32]. Here, we found that the CTD downregulated genes were associated with metabolic pathways, especially fatty acid metabolism and steroid biosynthesis. Moreover, we constructed the miRNA-target network of these hub genes. CTD regulates fatty acid metabolism thought targeting these potential miRNAs to downregulate identified hub genes enriching in fatty acid metabolism pathways. Finally, we constructed a CTD associated function and molecular regulation network of these hub genes involved in the autophagy, transcriptional misregulation and fatty acid metabolism pathways (Fig. 7A, B). Cantharidin inhibits tumor growth by integrating multiple pathway networks in HCC.

## Materials and methods

### Reagents and cells

Cantharidin was purchased from Sigma-Aldrich, China (C7632, purity ≥ 98%). H22 and HepG2 cells were obtained from the Cell Bank of the Chinese Academy of Sciences.

### Cell culture

All cells were cultured in a 6-well plate with DMEM (Dulbecco’s modified Eagle medium) (Gibco, USA) supplemented with 10% FBS (foetal bovine serum) (Gibco, USA), 100 µg/ mL streptomycin and 100 U/mL penicillin (Sigma, USA). All cells were maintained at 37 °C in a 5% CO_2_ atmosphere with saturated humidity.

### Establishment of animal model

The H22 mouse model of hepatocellular carcinoma ascites was established to confirm the antitumour function of cantharidin in vivo. H22 cells (5 × 10^7^ cells) were obtained and injected into BALB/c male mice, which were purchased from Shanghai Laboratory Animal Centre (Chinese Academy of Sciences, Shanghai, China). The axillary skin of the left forelimb of each mouse was disinfected with alcohol, and then 0.2 mL of diluted tumour cell suspension was injected subcutaneously. The tumour sizes were measured every three days. The model was considered to be successfully established when tumour tissue appeared in axillae of the mice after one week. Then, the H22 tumour-bearing mice were randomly divided into four groups, including the model group and three CTD treatment groups (n = 10 per group). Model group was treated with saline solution orally as control, treated groups that were therapy with different doses of CTD (low (0.25 mg/kg), middle (0.5 mg/kg) and high (1.0 mg/kg). The mice were weighed every 3 days during the drug treatment. After two weeks, the mice were fasted for 12 h and sacrificed by cervical dislocation. The tumours were obtained and weighed before washing in normal saline.

### Hematoxylin and eosin (H&E) staining

Tumour tissues were fixed with 4% paraformaldehyde. The tissues were dehydrated with 75% ethanol and embedded in paraffin. The samples were sliced into 4 μm-thick sections and dewaxed with xylene. Then, the sections were rehydrated through an ethanol gradient and stained using hematoxylin. Next, the sections were immersed in hydrochloric acid alcohol differentiation solution for 30 s, followed by eosin solution for 2 min. The sections were visualized and photographed using a digital scanning microscope.

### RNA-seq analysis

Total RNA was isolated by the TRIzol method (Invitrogen, Carlsbad, CA) according to the manufacturer’s instructions. The RNA samples were sequenced using the HiSeqTM 2500 system (Illumina). RNA-seq analysis was performed by Novogene Corporation Inc. (Beijing, China). The differences in the expression matrices generated from the sequencing data were analyzed with an R package. Gene expression profiles were constructed using the DESeq2 package. The differentially expressed genes identified by RNA-Seq were listed in the Supplementary materials.

### Gene ontology (GO) and Kyoto encyclopedia of genes and genomes (KEGG) analysis

To explore the underlying biological mechanism and pathways of the DEGs, GO analysis, KEGG analysis and Gene set enrichment analysis (GSEA) were used to confirm the functional and pathway based on the clusterProfiler package in the R platform [33]. GSEA hallmark gene sets were obtained from MSigDB (http://www.gsea-msigdb.org/gsea/msigdb). *p* < 0.05 was set as the significance threshold.

### Construction of protein-protein interaction (PPI) network

The PPI networks were constructed based on the STRING database and then visualized by Cytoscape software. The hub genes in these networks were confirmed with the Cytohubba plug-in in Cytoscape.

### Immune infiltration analysis

The expression of CTD related target genes was confirmed based on the TCGA LIHC database. The correlations between the expression of these genes and the infiltration of 25 types of immune cells were evaluated in LIHC based on GSCA (Gene Set Cancer Analysis) data.

### Prognostic analysis

To confirm the relationships between the CTD-related hub target genes and the prognosis of patients with liver cancer, the Kaplan-Meier Plotter was used to analyse the prognostic value of these genes in LIHC. *p* < 0.05 was considered to indicate a statistically significant differrnce.

### Real-time-PCR

Total RNA was extracted using RNA Plus reagent (Takara, China) and reverse transcribed using a PrimeScript RT Reagent Kit with gDNA Eraser (Takara, China). A SYBR Premix Ex Taq II (Takara, China) kit was used to determine the mRNA level of each gene. The assay was performed in the Thermal Cycler CFX6 System (Bio Rad, USA).

### Human protein atlas analysis

The protein levels of the target genes of CTD were assessed by immunohistochemistry based on the Human Protein Atlas, an online tool that contains data for protein expression in LIHC.

### Statistical analysis

All experiments were independently repeated three times. The data are summarized as the means ± standard deviations (SD). Student’s *t*-test was used to analysed the differences between two groups, while one-way ANOVA was performed to evaluate the statistical significance of differences among multiple groups, followed by Tukey’s post-hoc tests. The results were considered to be statistically significant when the value of *p* was < 0.05.

## Conclusion

This study set out to investigate and expand the possible functions of CTD in liver cancer therapy. We predicted the potential targets of CTD in HCC based on a comprehensive integrated analysis of its transcriptional profiles. The novel CTD target genes associated with autophagy, transcriptional misregulation and fatty acid metabolism were found, and network modules of theses pathways related-target genes were obtained. Moreover, miRNAs related to the regulation of fatty acid metabolism were confirmed, and a miRNA-mRNA network was constructed. In addition, these identified hub genes also play an important role in the immune response and tumour infiltration lymphocytes in HCC. Considered collectively, the integrated targets network of CTD will provide a novel way for exploring its function and mechanism in HCC therapy (Fig. 7). Interesting, metabolism-related genes, such as *BRCA1*, *MAPK3*, *CYP7A1*, and *AKR1C3*, serve as the core nodes of this network connecting other pathways, including phosphatase interacting and non-interacting auto/mitophagy pathways, TF dependent transcriptional regulation and immune response after CTD treatment in HCC. These findings are expected to guide to the application of CTD in the clinical treatment of liver cancer.

### Electronic supplementary material

Below is the link to the electronic supplementary material.

Supplementary Material 1

Supplementary Material 2

## Data Availability

The raw datasets analyzed during the current study are available in the GSA (Genome Sequence Archive) for human. GSA accession number was HRA004466 (subHRA006393). The database link was https://ngdc.cncb.ac.cn/search/?dbId=hra&q=HRA004466. All data generated or analysed during this study are included in this published article and its supplementary information files. The datasets used and analysed during the current study available from the corresponding author on reasonable request.
