# Supplementary figures and images for: Integrative analysis of the molecular signature of target genes involved in the antitumor effects of cantharidin on hepatocellular carcinoma
Source: BMC Cancer. 2023 Nov 28;23:1161. doi: 10.1186/s12885-023-11594-8 (PMC10685469; doi:10.1186/s12885-023-11594-8)

Figure S1. The chemical structure of cantharidin.


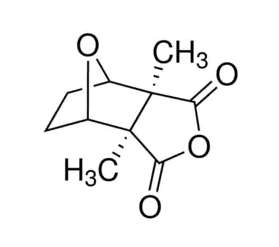

Supplement: Supplementary file 2 — Supplementary Material 2 [file 12885_2023_11594_MOESM2_ESM.docx]
